# Spatial variation and risk factors of the dual burden of childhood stunting and underweight in India: a copula geoadditive modelling approach

**DOI:** 10.1017/jns.2024.49

**Published:** 2024-09-26

**Authors:** Dhiman Bhadra

**Affiliations:** Operations and Decision Sciences Area, Indian Institute of Management Ahmedabad, Ahmedabad, Gujarat, India

**Keywords:** Copula geoadditive model, India, NFHS 5, Stunting, Underweight

## Abstract

India has one of the highest burdens of childhood undernutrition in the world. The two principal dimensions of childhood undernutrition, namely stunting and underweight can be significantly associated in a particular population, a fact that is rarely explored in the extant literature. In this study, we apply a copula geoadditive modelling framework on nationally representative data of 104,021 children obtained from the National Family Health Survey 5 to assess the spatial distribution and critical drivers of the dual burden of childhood stunting and underweight in India while accounting for this correlation. Prevalence of stunting, underweight and their co-occurrence among under 5 children were 35.37%, 28.63% and 19.45% respectively with significant positive association between the two (Pearsonian Chi square = 19346, P-value = 0). Some of the factors which were significantly associated with stunting and underweight were child gender (Adjusted Odds Ratio (AOR) = 1.13 (1.12) for stunting (underweight)), birthweight (AOR = 1.46 (1.64) for stunting (underweight)), type of delivery (AOR = 1.12 (1.19) for stunting (underweight)), prenatal checkup (AOR = 0.94 (0.96) for stunting (underweight)) and maternal short-stature (AOR = 2.19 (1.85) for stunting (underweight)). There was significant spatial heterogeneity in the dual burden of stunting and underweight with highest prevalence being observed in eastern and western states while northern and southern states having relatively lower prevalence. Overall, the results are indicative of the inadequacy of a “one-size-fits-all” strategy and underscore the necessity of an interventional framework that addresses the nutritional deficiency of the most susceptible regions and population subgroups of the country.

## Background

Childhood undernutrition is one of the most pressing public health crises facing the world today. It is estimated that nearly 50% of deaths in children under 5 are due to some form of undernutrition, while majority of these deaths occur in low and middle-income countries of South Asia and sub-Saharan Africa.^(1,2)^ Undernutrition is broadly categorised into stunting, wasting, underweight and vitamin and mineral deficiency. Of these, stunting, wasting and underweight are collectively known as child growth failure (CGF) and corresponds to low height-for-age, low weight-for-height and low weight-for-age, respectively. As per 2020 estimates, 149.2 million children under 5 are stunted, 45 million are wasted and 462 million are underweight. These correspond to 22%, 6.7% and 68.18% of all children under 5 globally.^(3)^ Due to its far-reaching negative consequences on childhood mortality, morbidity as well as on various aspects of health and wellbeing that continue into adulthood,^(4–11)^ childhood undernutrition is now considered a global health priority with various global targets being set by international bodies, all aimed towards its reduction and eventual eradication. For instance, the first of the six global nutrition targets set by the World Health Assembly Resolution 65.6 in 2012 specifies a 40% reduction in stuntedness among under-5 children by 2025.^(12–14)^ The aforementioned target is also part of the second Sustainable Development Goals (SDGs) set by the United Nations alongside ending hunger and all forms of malnutrition by 2030.

India has some of the highest rates of childhood undernutrition worldwide. As per current estimates, 31.7% of under 5 children are stunted, 18.7% are wasted while underweight incidence ranges from 39% to as high as 74% across the country. Despite the success of various government-run large-scale interventions, India is still way behind in achieving the aforementioned global nutritional targets.

The antecedents of childhood undernutrition are multifarious and there exists considerable literature on the same in the context of India.^(15–18)^ In all these studies, the different verticals of undernutrition are treated as independent and modelled separately. However, existing research has shown that these dimensions can be associated in a particular population and the degree of association can vary spatially.^(19–21)^ To our knowledge, rigorous analysis of the co-occurrence of various dimensions of childhood undernutrition is a relatively under-researched area, especially in the context of India. The current study endeavours to address this gap by modelling the dual burden of childhood stunting and underweight across the different states and union territories of India using nationally representative data from the National Family Health Survey-5 (NFHS-5) carried out during 2019–2021. Since rigorous modelling of multiple dimensions of childhood undernutrition is not possible in the conventional modelling framework, we have used a joint modelling approach in this study since it incorporates the implicit association between the various dimensions while modelling their co-occurrence. In doing so, it enables us to understand the spatial heterogeneity in their co-occurrence as well as in their association parameter.

Specifically this study provides four novel contributions to the literature. First, we identify the various drivers of childhood stunting and underweight while accounting for the implicit association between the two measures. Second, we use a flexible modelling approach to incorporate non-linear association between the determinants and the undernutrition indices. Third, we account for spatial variations of the association between stunting and underweight across the states of India. Finally, we quantify the joint likelihood of all possible combinations of stunting and underweight across all the states and union territories and produce spatial maps of the same. We implement these through a comprehensive modelling framework based on the copula geoadditive modelling approach.^(22,23)^ To our knowledge, this is one of the first studies that implements a joint modelling framework to analyse the co-occurrence of multiple dimensions of childhood undernutrition based on current nationally representative data from India. We hope that insights from this study will inform policymakers about regional variation in joint prevalence of childhood stunting and underweight and will aid in the formulation of effective nutritional interventions targeted towards the most susceptible regions and population subgroups of the country.

## Methods

### Data source

This study uses nationally representative data from the latest round of NFHS-5 carried out from June 2019 to April 2021. Like its previous counterparts, NFHS-5 provide estimates of key indicators of health and family welfare such as fertility, infant and child mortality, family planning practices and maternal and child health. The estimates are based on nationally representative data from 636,699 households, 724,115 women and 101,839 men selected using a two-stage stratified-cluster sampling scheme. The current study uses the children’s recode file of NFHS-5, which contains data on various nutrition and health indicators of children who were less than 5 years of age during the time of the interview. Data on maternal health and household characteristics for the sampled children were also available. Of the total of 232,920 children included in the survey, data on 104,021 children and their mothers were used for the analysis after necessary data cleaning. Informed consent was taken from all the participants either in the form of written or verbal means.

### Outcomes and covariates of interest

The outcomes of interest were childhood stunting and underweight. Anthropometric measures of children’s height-for-age and weight-for-age *Z*-scores (HAZ and WHZ) were used to construct indicators of these indices. Specifically, children whose standardised HAZ and WAZ scores were below –2 were labelled as stunted and underweight respectively.^(24,25)^ This corresponds to having height-for-age and weight-for-age values less than –2 standard deviations from the World Health Organization child growth standards median.^(26)^ Children with scores more than 6 or less than –6 for either of these measures were treated as outliers and dropped. For children aged 2 years or below, height was measured using Seca 417 infantometer while for children aged 24–59 months, a Seca 213 stadiometer was used. Weight was measured using Seca 874 digital scale. Thus the dependent variables were binary with categories “stunted” and “not stunted” and “underweight” and “not underweight” respectively. Having said that, the main purpose of this study is to explore the spatial variation and determinants of the dual burden of malnutrition among Indian children characterised by the co-occurrence of stunting and underweight.

The antecedents for stunting and underweight for each child included age of the child (in months), age of mother at first birth (in years), duration of breastfeeding (in months), source of drinking water (1 if improved, 0 otherwise), quality of sanitation (1 if improved, 0 otherwise), prenatal care from doctor (1 if yes, 0 otherwise), whether child had low birth- weight (less than 2500 gms of weight at birth) (1 if yes, 0 if no), number of children in the household aged 5 years or below, child gender (1 for male, 2 for female), gender of household head (1 for male, 2 for female), mode of delivery (1 for caesarean, 0 otherwise), short stature of mother (height less than 145 cm) (1 if yes, 0 if no), place of residence (0 if urban, 1 if rural), whether mother is underweight (body mass index less than 20) (1 if yes, 0 if no), maternal educational attainment (0 if no education, 1 if primary education, 2 if secondary education and 3 if higher secondary education with 0 being the baseline), household wealth quintile (1 if poorest, 2 if poorer, 3 if middle, 4 if richer and 5 if richest with 1 being the baseline), caste (1 if scheduled caste, 2 if scheduled tribe, 3 if other backward classes and 4 if none of those with 1 being the baseline), perceived size at birth (1 if very large, 2 if larger than average, 3 if average, 4 if smaller than average and 5 if very small with 1 being the baseline), maternal anaemia level (1 if haemoglobin(Hb) 8 g/dl, 2 if 8 Hb 11, 3 if 11 Hb 12 and 4 if Hb 12 with 1 being the baseline) and childhood anaemia (1 if haemoglobin(Hb) 7 g/dl, 2 if 7 ≤ Hb <10, 3 if 10≤ Hb < 11 and 4 if Hb ≥ 11 with 1 being the baseline).

### Copula geoadditive models

Our empirical strategy hinges on a framework that enables the joint modelling of two binary responses, namely childhood stunting and underweight while accommodating the implicit association between the two. This is achieved using a copula geoadditive modelling framework that incorporate spatial heterogeneity in the stunting-underweight association as well as flexible non-linear functions of covariates.^(22,27)^ In this setup, the dependence structure between the two responses is modelled using a special class of functions that enable flexible specification of the marginal models of each of the responses separately from that of the joint distribution governing their dependence structure. These functions are known as copulas.^(28)^ Although a relatively new concept, copulas have been extensively used for modelling association between diverse class of responses across multiple fields. Applications range from modelling mixed binary-continuous data,^(29)^ continuous and discrete longitudinal data,^(30)^ censored data^(31)^ and count data.^(32)^ Copula models have been used in finance and insurance,^(33,34)^ forestry and environment^(35)^ and marketing^(36)^ and public health.^(37,38)^ Excellent reviews of copula models are provided by Trivedi and Zimmer (2007) and Genest (2007).

Let *Y_is_* and *Y_iu_* be the stunting and underweight status of the *i* ^*th*^ child such that *Y_is_ =* 1 (0) if the child is stunted (not stunted) and *Y*_*iu*_ = 1 (0) if the child is underweight (not underweight). The copula framework enables the specification of the joint probability of the *i* ^*th*^ child being stunted as well as underweight as

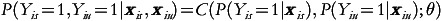




where ***x***_*is*_ and ***x***_*iu*_ are the attribute vectors corresponding to stunting and underweight respectively. Here *C* ⋮ [0,1]^2^ → [0,1] is a two-place copula function while *θ* is the copula parameter that quantifies the association between stunting and underweight prevalence.^(23)^ The marginal probabilities of being stunted or underweight i.e *P (Y*_*is*_ = 1| ***x***_*is*_ *)* or *P(Y*_*iu*_ = 1| ***x***_*iu*_ *)* is parameterised using the following latent variable representation

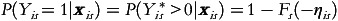




where Y*_is_ is a continuous latent variable expressible as Y*_is =_ *η*_*is +*_ *ε*_*is*_, *η*_*is*_ being the linear predictor consisting of linear, non-linear as well as structured and unstructured spatial effects while *ε*_*is*_ is a white noise error. *F*_*s*_ (−*η*_*is*_) is the cumulative distribution function of the error and determines the structure of the marginal model linking the stunting indicator *Y*_*is*_, to the corresponding linear predictor. The flexibility of the copula approach lies in the fact that *F(.)* can correspond to a broad class of univariate distributions (Gaussian, logistic, Gumbel for instance) depending on the assumed distributional form for the error term. For instance, a standard normal distributional assumption for *ε*_*is*_ would lead to a probit specification for the corresponding marginal model. A similar setup can be replicated for the underweight indicator, *Y*_*iu*_.

The flexibility of the copula geoadditive framework lies in its ability to incorporate a diverse class of effects in the marginal model specifications. In our setup, we accommodate the following four types of effects in the marginal models for stunting and underweight:

(i) regular fixed effects of the categorical variables and of those continuous variables which are linearly related to the response; (ii) flexible non-linear effects for variables which have a curvilinear association with the response; (iii) within-region (unstructured) spatial effects to account for the presumed similarity in stunting and wasting prevalence among children residing in the same region and lastly; (iv) between-region (structured) spatial effects to account for the assumed dependence in stunting and wasting prevalence among children residing in adjacent regions. The non-linear effects are estimated by thin-plate regression splines while the structured spatial effects are estimated by Markov random field smoother which is based on the neighbourhood structure of the regions.^(23,29)^ For the purpose of modelling, we used the Frank copula to model the dependence between stunting and underweight and the logit specification for the marginal models of stunting and underweight. We chose the Frank copula for its flexibility in accommodating the full range of correlation values for the two responses.^(39)^ The Frank copula has the following expression

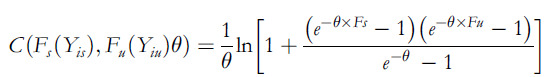




In addition to the marginal models of stunting and underweight, we modelled the copula parameter, *θ* with respect to the state boundaries in order to capture any spatial variation in the association between stunting and underweight across the states and union territories of India. This may enable the identification of particular states where stunting and underweight prevalences are strongly or weakly associated, which, in turn, can provide valuable insights to policymakers on the need for region-specific interventions. For ease of interpretation, the copula parameter was transformed to Kendall’s tau correlation coefficient, *τ* which is a measure of the degree of concordance between two variables.^(23)^ Finally, we create joint probability maps depicting the between-state variation in the co-occurrence of stunting and underweight.

Analysis was carried out using the R package *GJRM* (Generalized Joint Regression Modelling)^(23,40)^ while mapping was carried out in QGIS 3.22 using shapefiles freely obtainable from the Spatial Data Repository maintained by the DHS programme (https://spatialdata.dhsprogram.com/boundaries). All estimates have been weighted using the sampling weights provided in the NFHS-5 data file. The standard errors of all the estimates have been suitably adjusted to account for the multistage cluster sampling carried out in the survey.

## Results

We present our results in four stages. First, we discuss the prevalence and distribution of stunting and underweight across various socio-economic and demographic subgroups as identified by the child, maternal and household-specific attributes. We next present the linear as well as non-linear effects of the various covariates on the likelihood of stunting and underweight as derived from the marginal models. Next, we discuss the unstructured and structured spatial effects of stunting, underweight and their association across India. Finally, we elaborate on the insights obtained from joint probability maps of the dual burdens of stunting and underweight.

### Stunting and underweight prevalence

Overall, 35.37% of the sampled children were stunted, 28.63% were underweight and 19.45% were both stunted and underweight. The prevalence of all the three conditions was higher among males (36.8% stunted, 29.9% underweight, 20.5% both) than females (33.7%, 27.2%, 18.2%). Children belonging to the scheduled caste category had the highest prevalence of all the three conditions (39.1%, 32.1%, 22.2%) followed by those in the scheduled tribe (38.6%, 30.3%, 21.1%), other backward classes (35.3%, 29.3%, 19.9%) and unreserved categories respectively (27.5%, 21.3%, 13.5%). Caesarean children had lower incidence of all the three conditions (29.2%, 22.3%, 14.5%) compared to those born through normal means (37.2%, 30.5%, 20.9%). Children whose perceived size at birth was “very small” had the highest incidence of all the three conditions (46.4%, 42.3%, 30.7%). Similarly, severely anaemic children had the highest incidence of the three conditions (46.1%, 36.5%, 27.8%) while the incidence gradually reduced with improvements in anaemia status. Finally, children having low birthweight had higher incidence of the three ailments (44.1%, 39.4%, 28.4%) compared to those having normal birthweight (33.7%, 26.6%, 17.7%). As far as maternal attributes are concerned, prevalence of each of the three conditions was substantially higher among children whose mothers had short stature (54.4%, 44.2%, 34.6%) or were underweight (42.2%, 37.8%, 26.4%) compared to those whose mothers had normal physique. Children whose mothers did not receive prenatal treatment from a certified physician had higher incidence of the conditions (39.2%, 32.5%, 22.6%) compared to those whose mothers received such care (33.1%, 26.4%, 17.6%). Children whose mothers had at least a higher secondary education had the lowest incidence of all the three conditions (23.7%, 17.4%, 10.3%) while the incidence steadily increased with lower maternal educational attainment. Highest incidence of the three conditions was observed among children whose mothers had severe anaemia (43.0%, 35.7%, 25.5%) followed by those whose mothers were moderately anaemic (38.0%, 31.1%, 21.6%), mildly anaemic (34.7%, 28.9%, 19.5%) and non-anaemic respectively (33.5%, 26.3%, 17.6%). As far as locational attributes were concerned, incidence of all the three ailments was lower among children hailing from urban regions (29.6%, 23.5%, 15.1%) compared to those from rural areas (37.0%, 30.1%, 20.7%). Children belonging to the richest households had the lowest incidence of all the three ailments (23.2%, 16.7%, 10.0%) while those belonging to the poorest households had the highest incidence (46.2%, 39.8%, 28.6%). Finally, lower incidence of the ailments were observed in households with improved sanitation facility (32.8%, 25.8%, 17.1%) and those with availability of clean drinking water (35.3%, 28.6%, 19.4%) compared to households lacking such facilities (Table 1).

**Table 1.** Prevalence of stunting, underweight, and their co-occurrence among under 5 children

	Stunting		Underweight		Stunting & underweight	
	Proportion/Mean	Std. Dev	Proportion/Mean	Std. Dev	Proportion/Mean	Std. Dev
Overall	0.354	0.478	0.286	0.452	0.194	0.396
Child characteristics						
Gender						
Male	0.368	0.482	0.299	0.458	0.205	0.404
Female	0.337	0.473	0.272	0.445	0.182	0.386
Caste						
Scheduled caste	0.391	0.488	0.321	0.467	0.222	0.415
Scheduled tribe	0.386	0.487	0.303	0.460	0.211	0.408
Other backward classes	0.353	0.478	0.293	0.455	0.199	0.399
Non-reserved/General	0.275	0.447	0.213	0.410	0.135	0.342
Mode of delivery						
Caesarean	0.292	0.454	0.223	0.416	0.145	0.352
Normal	0.372	0.483	0.305	0.460	0.209	0.406
Perceived size at birth						
Very large	0.352	0.477	0.282	0.450	0.191	0.393
Larger than average	0.337	0.473	0.264	0.441	0.179	0.383
Average	0.348	0.476	0.279	0.448	0.188	0.391
Smaller than average	0.410	0.492	0.358	0.479	0.254	0.436
Very small	0.464	0.499	0.423	0.495	0.307	0.461
Anaemia						
Severe	0.461	0.499	0.365	0.482	0.278	0.448
Moderate	0.400	0.490	0.318	0.465	0.224	0.417
Mild	0.343	0.475	0.285	0.452	0.188	0.391
Normal	0.299	0.458	0.243	0.429	0.157	0.364
Low birthweight						
Yes	0.441	0.496	0.394	0.489	0.284	0.451
No	0.337	0.473	0.266	0.442	0.177	0.382
Age	29.95	14.57	30.58	14.95	31.19	14.52
Breastfeeding duration	20.09	12.41	20.38	12.69	20.87	12.73
Maternal characteristics						
Age at first birth	21.17	3.59	21.11	3.58	20.96	3.51
Prenatal care by doctor						
Yes	0.331	0.471	0.264	0.441	0.176	0.381
No	0.392	0.488	0.325	0.468	0.226	0.418
Shortstature						
Yes	0.544	0.498	0.442	0.497	0.346	0.476
No	0.330	0.470	0.267	0.442	0.176	0.380
Underweight						
Yes	0.422	0.494	0.378	0.485	0.264	0.441
No	0.319	0.466	0.239	0.427	0.159	0.366
Education						
No	0.453	0.498	0.390	0.488	0.282	0.450
Primary	0.425	0.494	0.346	0.476	0.246	0.431
Secondary	0.338	0.473	0.270	0.444	0.179	0.384
Higher secondary	0.237	0.425	0.174	0.379	0.103	0.305
Maternal characteristics						
Anaemia						
Severe	0.430	0.495	0.357	0.479	0.255	0.436
Moderate	0.380	0.485	0.311	0.463	0.216	0.411
Mild	0.347	0.476	0.289	0.453	0.195	0.396
Normal	0.335	0.472	0.263	0.440	0.176	0.381
Household characteristics						
Region						
Urban	0.296	0.457	0.235	0.424	0.151	0.358
Rural	0.370	0.483	0.301	0.459	0.207	0.405
Household head						
Male	0.351	0.477	0.287	0.452	0.194	0.396
Female	0.366	0.482	0.281	0.449	0.196	0.397
Household characteristics						
Wealth						
Poorest	0.462	0.498	0.398	0.489	0.286	0.452
Poorer	0.399	0.490	0.323	0.468	0.227	0.419
Middle	0.343	0.475	0.270	0.444	0.180	0.385
Richer	0.284	0.451	0.226	0.418	0.139	0.346
Richest	0.232	0.422	0.167	0.373	0.100	0.300
Toilet facility						
Improved	0.328	0.469	0.258	0.438	0.171	0.377
Unimproved	0.443	0.497	0.383	0.486	0.274	0.446
Drinking water						
Available	0.353	0.478	0.286	0.452	0.194	0.396
Not available	0.369	0.482	0.295	0.456	0.200	0.400
Under 5 child	1.56	0.76	1.56	0.76	1.58	0.76

In order to assess the strength of association between stunting and being underweight, we performed a Pearsonian chi-square test which yielded a P-value of 0 indicating strong positive association between the two. This validates the necessity of joint modelling of the ailments using the copula modelling framework.

### Linear covariate effects

Adjusted for all other variables, boys had a significantly higher odds of being stunted as well as underweight compared to girls (AOR = 1.13, 95% CI 1.10–1.16 for stunting and AOR = 1.12, 95% CI = 1.09–1.16 for underweight). Similarly, children born through caesarean section had a significantly lower odds of being stunted and underweight compared to those born through normal delivery (AOR = 0.89, 95% CI = 0.86–0.92 for stunting and AOR = 0.84, 95% CI = 0.81–0.87 for underweight). Children who had low birth weight had a significantly higher odds of both the conditions (AOR = 1.46, 95% CI = 1.41–1.52 for stunting and AOR = 1.64, 95% CI = 1.58–1.71 for underweight). Interestingly, children hailing from rural regions had significantly lower odds of both stunting and underweight (AOR = 0.94, 95% CI = 0.91–0.97 for stunting and AOR = 0.91, 95% CI = 0.87–0.94 for underweight). As far as maternal attributes are concerned, children whose mothers received prenatal checkup from a doctor were significantly less likely to be either stunted or underweight than those whose mothers did not receive such treatment (AOR = 0.94, 95% CI = 0.92–0.98 for stunting and AOR = 0.96, 95% CI = 0.93–0.99 for underweight). Likewise, children whose mothers were short-statured had significantly higher odds of being stunted or underweight (AOR = 2.19, 95% CI = 2.1–2.28 for stunting and AOR = 1.85, 95% CI = 1.78–1.93 for underweight) and similar effects were observed for underweight mothers (AOR = 1.30, 95% CI = 1.26–1.34 for stunting and AOR = 1.57, 95% CI = 1.53–1.62 for underweight) as well. Higher maternal educational attainment, higher household wealth quintile and availability of clean drinking water and improved sanitation facilities on-premise correspond to significantly lower odds of stunting and underweight while higher severity of maternal and childhood anaemic status correspond to significantly higher odds of both the conditions. Newborns with perceived birthsize “smaller than average” and “very small” have a significantly higher odds of being stunted as well as underweight compared to the complement groups. As far as caste is concerned, children belonging to other backward classes and non-reserved (general) caste have significantly lesser odds of being stunted as well as underweight compared to those belonging to scheduled castes. However, children belonging to the scheduled tribes have a significantly higher odds of being underweight compared to those from the scheduled castes. Finally, the odds of stunting and underweight prevalence are significantly higher in households with a higher number of under-5 children. This is indicative of the benefits of family planning measures in reducing the prevalence of childhood undernutrition (Table 2).

**Table 2.** Estimated odds ratios and 95% confidence intervals of stunting and underweight

	Stunting OR (95% CI)	Underweight OR (95% CI)
Child characteristics		
Gender (Reference category: male)		
Female	0.89^***^ (0.86–0.91)	0.89^***^ (0.86–0.91)
Mode of delivery (Reference category: normal delivery)		
Caesarean	0.89^***^ (0.86–0.92)	0.84^***^ (0.81–0.87)
Birthweight (Reference category: high)		
Low	1.46^***^ (1.41–1.52)	1.64^***^ (1.58–1.71)
Caste (Reference category: scheduled caste)		
Scheduled tribe	0.97 (0.92–1.02)	1.06^**^ (1.00–1.11)
Other backward classes	0.95^***^ (0.92–0.99)	0.96^**^ (0.92–0.99)
General	0.82^***^ (0.78–0.85)	0.82^***^ (0.78–0.86)
Perceived size at birth (Reference category: very large)		
Larger than average	0.95 (0.89–1.02)	0.99 (0.93–1.06)
Average	1.04 (0.98–1.09)	1.11^***^ (1.05–1.17)
Smaller than average	1.18^***^ (1.11–1.27)	1.27^***^ (1.18–1.37)
Very small	1.21^***^ (1.09–1.34)	1.54^***^ (1.39–1.69)
Anaemia (Reference category: severe)		
Moderate	0.78^***^ (0.71–0.85)	0.74^***^ (0.67–0.81)
Mild	0.63^***^ (0.58–0.69)	0.67^***^ (0.61–0.74)
Normal	0.54^***^ (0.49–0.59)	0.58^***^ (0.53–0.64)
Maternal characteristics		
Education (Reference category: no education)		
Primary	0.94^***^ (0.89–0.98)	0.91^***^ (0.87–0.96)
Secondary	0.81^***^ (0.78–0.84)	0.81^***^ (0.78–0.84)
Higher secondary	0.66^***^ (0.62–0.70)	0.67^***^ (0.63–0.71)
Shortstature (Reference category: no)		
Yes	2.19^***^ (2.10–2.28)	1.85^***^ (1.78–1.93)
Prenatal care by doctor (Reference category: no)		
Yes	0.94^***^ (0.92–0.97)	0.96^***^ (0.93–0.99)
Underweight (Reference category: no)		
Yes	1.30^***^ (1.26–1.34)	1.57^***^ (1.53–1.62)
Anaemia (Reference category: severe)		
Moderate	0.90^**^ (0.82–0.98)	0.89^**^ (0.81–0.98)
Mild	0.85^***^ (0.78–0.94)	0.88** (0.80–0.97)
Normal	0.87^***^ (0.79–0.95)	0.87^***^ (0.79–0.96)
Household characteristics		
Residence (Reference category: urban)		
Rural	0.94^***^ (0.91–0.97)	0.91^***^ (0.87–0.94)
Household head (Reference category: male)		
Female	1.04^*^ (1–1.08)	0.93^***^ (0.89–0.97)
Wealth (Reference category: poorest)		
Poorer	0.89^***^ (0.85–0.93)	0.86^***^ (0.82–0.90)
Middle	0.78^***^ (0.74–0.82)	0.72^***^ (0.68–0.75)
Richer	0.64^***^ (0.60–0.67)	0.62^***^ (0.59–0.66)
Richest	0.56^***^ (0.53–0.60)	0.50^***^ (0.47–0.54)
Toilet facility (Reference category: unimproved)		
Improved	0.92^***^ (0.89–0.96)	0.95^***^ (0.92–0.99)
Drinking water (Reference category: not available)		
Available	1.02^***^ (1.10–1.18)	1.08^**^ (1.00–1.16)

* Significant at the 10% level.

** Significant at the 5% level.

*** Significant at the 1% level.

### Non-linear covariate effects

**Fig. 1.**
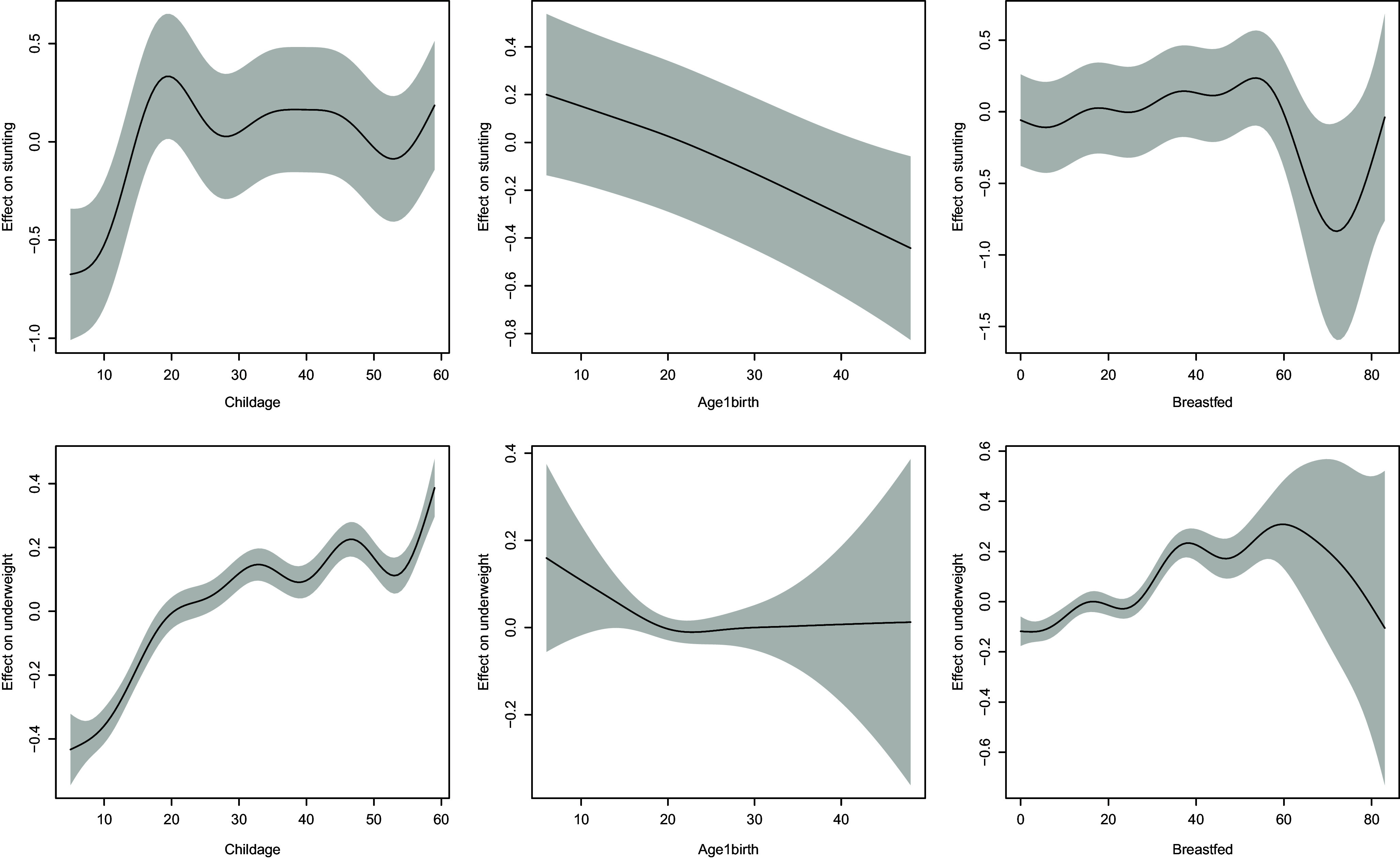
Estimated non-linear effects of child age, maternal age at first birth and duration of breastfeeding on the likelihood of stunting (top row) and underweight (bottom row). Shaded regions correspond to 95% confidence bands.

Table 3 and Fig. 1 depict the significance of the non-linear covariate effects corresponding to child age, maternal age at first birth and duration of breast feeding. It is apparent that these covariates have significant non-linear association with the incidence of stunting while child age and duration of breastfeeding have significant non-linear association with underweight.

**Table 3.** Statistical significance of non-linear effects of child age, maternal age at first birth and breastfeeding duration on childhood stunting and underweight in India

	Stunting		Underweight	
	Chi-square	P -value	Chi-square	P -value
Age of child (months)	739.49	< 0.001	422.55	< 0.001
Age at first birth	54.62	< 0.001	5.46	0.16
Duration of breastfeeding (months)	90.44	< 0.001	156.14	< 0.001

### Structured and unstructured spatial effects

**Fig. 2.**
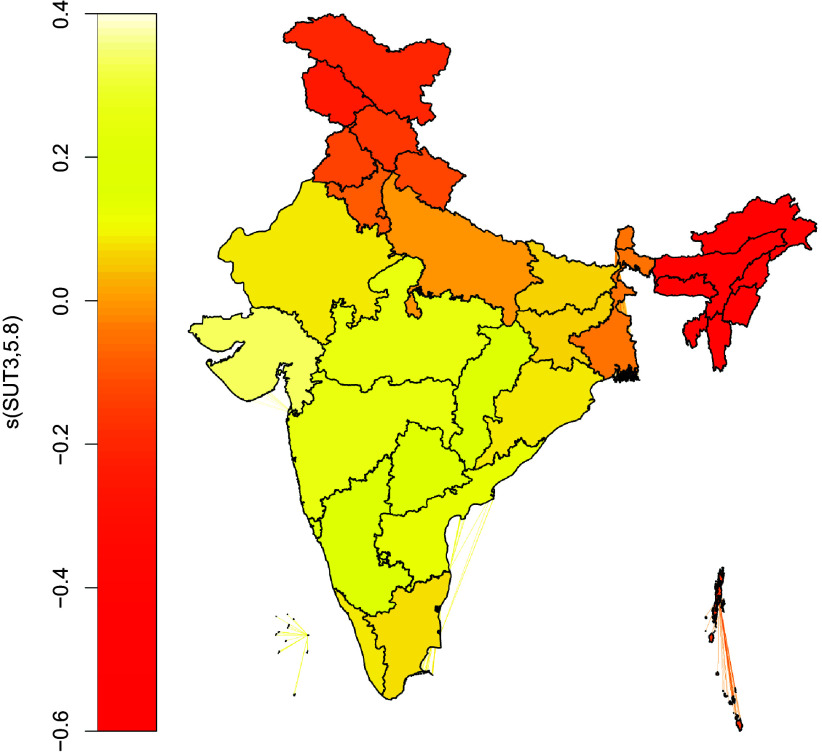
Structured spatial effect for childhood underweight.

Table 4 depicts the structured and unstructured spatial effects of stunting and underweight. The unstructured spatial effects are significant at 5% level implying considerable within-state variation in the prevalence of stunting and underweight. The structured spatial effect of underweight is also significant at 5% level implying considerable between-state variation in its prevalence. Figure 2 depicts the structured spatial effect of underweight across India. Specifically, childhood underweight seems to have the lowest prevalence in the north-eastern states followed by states in northern India while the prevalence is highest in central and western India. The copula parameter was found to be significant at 1% level implying significant spatial heterogeneity in the stunting-underweight association across states and union territories. The Kendall’s tau coefficient was 0.4 with a 95% confidence interval of (0.384, 0.418) implying moderately strong stunting-underweight association across India.

**Table 4.** Statistical significance of structured and unstructured spatial effects of stunting and underweight among under 5 children in India

	Stunting		Underweight	
	Chi-square	P -value	Chi-square	P -value
Unstructured spatial effect	418.20	< 0.001	447.13	< 0.001
Structured spatial effect	2.31	0.545	19.88	< 0.01

### Joint probability maps

**Fig. 3.**
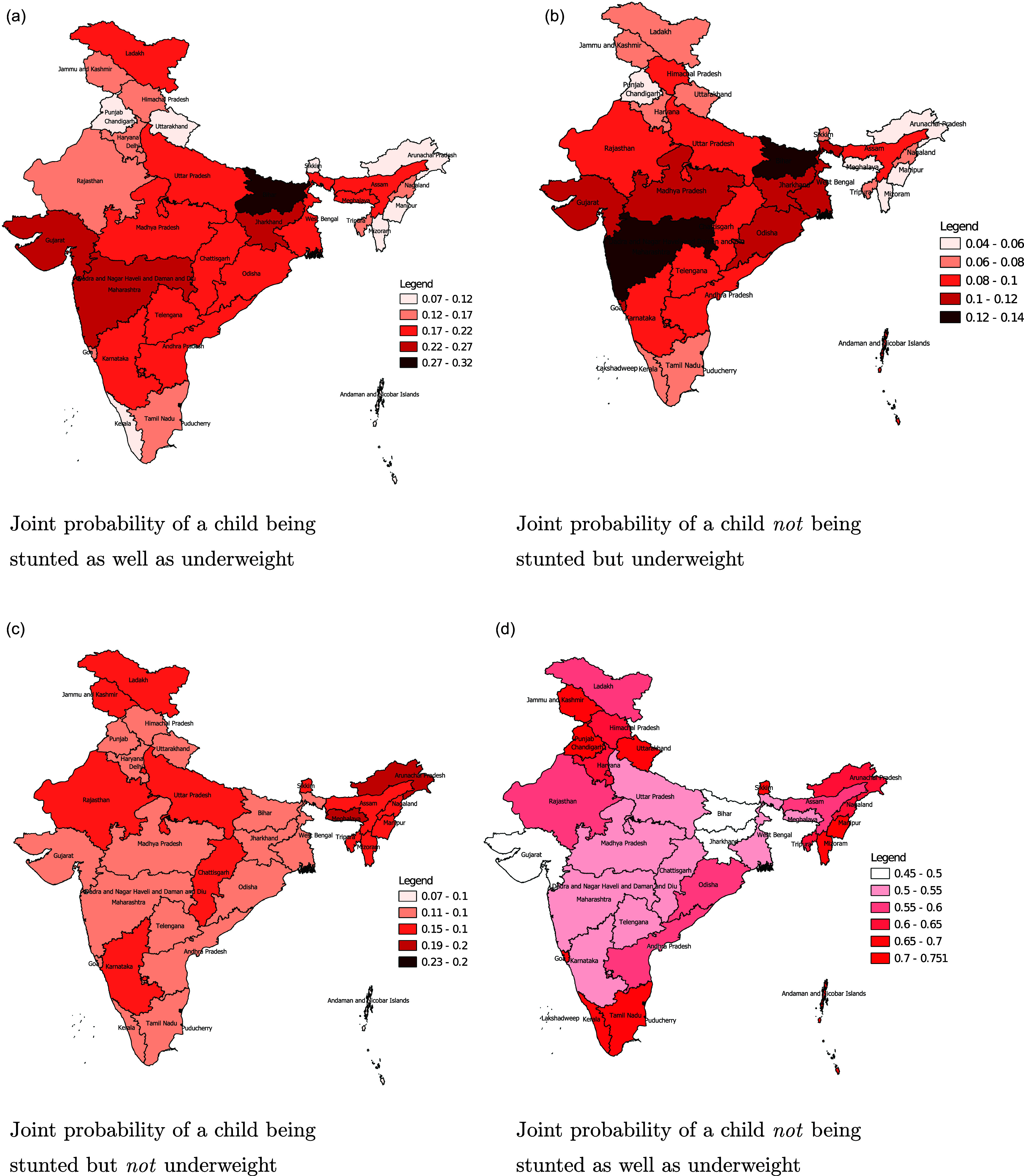
Joint probability maps depicting the co-occurrence of stunting and underweight across states and union territories of India.

In order to better understand the spatial variation in the dual burden of stunting and underweight, we produced maps depicting the joint probabilities of a child being (i) neither stunted nor underweight, (ii) stunted but not underweight, (iii) underweight but not stunted and (iv) stunted as well as underweight in Fig. 3. The highest probability of co-occurrence of stunting and underweight was observed in the relatively impoverished eastern state of Bihar closely followed by the adjacent state of Jharkhand as well as in the considerably wealthier western states of Gujarat and Maharashtra. This apparent “anomaly” is indicative of the fact that economic development may not always go hand-in-hand with improvements in maternal and child health. The lowest co-occurrence of stunting and underweight is observed in the agriculturally rich northern state of Punjab, the thinly populated mountainous state of Uttarakhand, union territories of Andaman and Nicobar and Lakshadweep islands as well as in the progressive southern state of Kerala and north-eastern states of Manipur and Mizoram. Along similar lines, children belonging to Bihar, Jharkhand and Gujarat have the lowest probabilities of being neither stunted nor underweight while those hailing from southern states of Kerala, Tamilnadu, northern states of Punjab, Uttarakhand and Jammu and Kashmir and eastern states of Mizoram, Manipur, Arunachal Pradesh and Sikkim have the highest propensity of being free of both these ailments.

A few insights can be derived from these maps. Firstly, there is noticeable spatial heterogeneity in the co-occurrence of stunting and underweight across the states of India. Secondly, there is moderately strong pan-India association between childhood stunting and being underweight. Lastly, the drivers of stunting and underweight may not necessarily overlap as indicated by the moderately strong probability of the occurrence of one ailment in the absence of another in some states. This points to the necessity of incorporating regional attributes for effective policy formulation and impact maximisation.

## Discussion

This study relates to the modelling of the dual burden of childhood stunting and underweight and its antecedents in India. This is achieved using a copula geoadditive modelling framework^(23,29)^ which accounts for the implicit association between the two dimensions of undernutrition as well as their spatial heterogeneity across the country. To the best of our knowledge, this is one of the first and the largest studies carried out on nationally representative data from India that employs a joint modelling framework to analyse the dual burden and geographical variation of two major dimensions of childhood undernutrition.

Marginal prevalence of stunting and underweight were 35.37% and 28.63% while their dual prevalence was 19.45%. Stunting and underweight had significant positive correlation at the pan-India level which called for the necessity of their joint modelling. Childhood underweight had significant between-state spatial variation while the variation was lesser for childhood stunting. This partly corroborated the findings from previous studies which have demonstrated significant spatial variation in both childhood stunting and underweight across various sub-national boundaries of India.^(16,18,41–43)^ Interestingly, childhood underweight has higher prevalence in the relatively wealthier western states of India while it is less prevalent in the north-eastern and northern parts of this country. Spatial maps of the joint probabilities of stunting and underweight depicted higher co-occurrence of stunting and underweight both in the poorer eastern states of Bihar and Jharkhand as well as in the relatively wealthier western states of Gujarat and Maharashtra. Lower prevalence was observed in relatively prosperous, less populated and developed states of the northern, north-eastern and southern parts of the country. This is an interesting finding since it is indicative of the fact that economic growth may not always go hand-in-hand with improvements in maternal and child health. The joint prevalence of underweight but not stunting was uniformly low across India which validated their close association. The co-occurrence of stunting but not of underweight is lowest in the Andaman and Nicobar Islands and highest in Arunachal Pradesh, Meghalaya and Lakshadweep islands. This is indicative of the presence of other drivers of stunting apart from underweight in these regions. To our knowledge, these insights are unavailable in any previous studies on childhood undernutrition in India.

Various drivers at the child, maternal and household levels had significant association with the prevalence of stunting and underweight. As far as the child-specific drivers were concerned, girls had a significantly lesser odds of being stunted as well as underweight compared to boys. This is well aligned with previous studies from developing countries.^(44–46)^ Children having low birthweight had significantly higher odds of stunting as well as underweight compared to those having normal birthweight. This is broadly consistent with that observed in previous studies.^(46,47)^ Children who were perceived to be very small or smaller than average during birth had a significantly higher odds of stunting and underweight compared to those whose perceived birth size was normal. This is well established in the undernutrition literature as well.^(44,45)^ Children born through caesarean section had significantly lesser odds of being either stunted or underweight compared to those born through normal delivery. Similarly, severity of childhood anaemia had a direct association with undernutrition with higher severity corresponding to higher odds of both forms of undernutrition. Finally, children belonging to unreserved categories had a significantly lesser odds of both stunting and underweight compared to those belonging to scheduled castes. To our knowledge, these observations are fairly new specifically in the context of joint analysis of stunting and underweight of current nationally representative data from India.

Children whose mothers were underweight or short-statured have significantly higher odds of being both stunted or underweight compared to children of healthy mothers. Maternal educational attainment had a significant negative influence on the incidence of childhood undernutrition with higher levels of attainment leading to significantly lower odds of both forms of undernutrition markers. These results align with those from previous studies on India and other developing countries.^(16,44,45,47–49)^ Children belonging to non-anaemic mothers had a significantly lower odds of being either stunted or underweight compared to those belonging to severely anaemic mothers. This extends the findings of previous studies which observed similar association patterns between maternal anaemia and childhood stunting.^(17)^ Finally, mothers who have received prenatal care from a registered physician have a significantly lower odds of having a stunted or underweight baby compared to those who did not have access to such care. This improves upon the findings from previous studies which have shown a positive impact of receiving prenatal care in reducing the likelihood of childhood stunting.^(47)^

Children from rural areas had a significantly lower odds of being either stunted or underweight compared to those from urban areas. This is in divergence with findings from previous studies which reported lesser odds of stunting for children hailing from urban localities.^(17,45,47)^ However, this is consistent with a previous study on childhood undernutrition in Bangladesh which depicted higher prevalence of stunting among children in urban localities than their counterparts in rural regions.^(44)^ One reason for this can be the significantly lower pollution and healthier environmental conditions prevailing in rural parts of India as opposed to the urban metros many of which suffer from some of the highest levels of pollution in the world. Another important factor can be the improved implementation and better penetration of various government schemes aimed at meeting the nutritional requirements of susceptible population subgroups primarily located in the rural areas. Households having a female head have a significantly lower odds of having underweight children although no such association exists for stunting. Finally, households belonging to higher wealth quintiles and those having better access to clean drinking water and improved sanitation facilities have significantly lesser odds of having a stunted or underweight child. These are broadly consistent with findings from previous studies carried out in developing countries.^(16,17,44,46,48,49)^

As far as continuous predictors were concerned, child age, maternal age at first birth and breastfeeding duration had significant non-linear association with the likelihood of stunting as well underweight. Specifically, children who are breastfed beyond 5 years seem to have lower odds of stunting as well as underweight while higher maternal age at first birth has a positive impact in reducing the onset of stunting. To our knowledge, these are relatively novel findings in the context of India and add to those obtained from previous studies on childhood undernutrition in low and middle-income countries.^(47,48,50)^

The key strength of our study is that it is one of the first and largest studies in India that jointly models the co-occurrence of childhood stunting and underweight while accounting for their spatial heterogeneity across sub-national boundaries. Previous studies that jointly modelled multiple undernutrition indicators for India^(37,38)^ were based on much smaller sample sizes and also accounted for a smaller number of predictors compared to the current study. Having said that, this study is not free from limitations which may provide pointers for future research. First and foremost, the study uses cross-sectional data from a single round of NFHS thus limiting the scope for causal inference. Second, environmental predictors such as cluster height, vegetation index and land surface temperature have not been accounted for in the modelling framework, which, if done, can further our understanding of the impact of environmental drivers on the co-occurrence of childhood stunting and undernutrition. Third, socio-economic and demographic variables can be incorporated into the model for the copula parameter which would better help us in understanding how the stunting-underweight association varies across socio-economic strata.

## Conclusion

In conclusion, the current study provides interesting data-driven insights about regional variation in the dual occurrence of childhood stunting and underweight based on the latest nationally representative data from India. The resulting spatial maps can help in the identification of states with high propensity of both stunting and underweight as well as high propensity of one and low propensity of the other ailment. This study also identifies various risk factors at the child, maternal and household levels which have significant association with stunting and underweight as well as the exact nature of the association pattern.

Overall, the results of this study are indicative of the fact that a “one-size-fits-all” strategy may be sub-optimal for a vast and diverse country like India and calls for a more nuanced, region-specific intervention framework to effectively tackle the scourge of childhood undernutrition. The considerable within and between-state heterogeneity in malnutrition also points to the importance of designing state or even region-specific nutritional intervention plan that accounts for the specific needs and characteristics of that state or region. One such example is the Rajmata Jijau Mother-Child Health and Nutrition Mission launched in the western state of Maharashtra in 2005 which has been credited with successfully reducing stunting rates in children aged under 2 years, from 44% in 2005 to 22.8% in 2012. One can also examine similar success stories from Brazil, Peru and Bolivia.^(14)^ These case studies stand testament to the importance of effective policy formulation, careful implementation and diligent monitoring and supervision as weapons in the fight towards ending all forms of hunger and malnutrition specifically in high-burden countries like India.

## Abbreviations

**NFHS:** National Family Health Survey; **CGF:** child growth failure; **SDG:** sustainable development goals; **AOR:** adjusted odds ratio; **GJRM:** generalised joint regression modelling; **DHS:** Demographic Health Survey; **HAZ:** height-for-age; **WHZ:** weight-for-height; **WAZ:** weight-for-age; **IIPS:** Indian Institute of Population Sciences.
